# High-salt diet promotes atopic dermatitis by partially enhancing intestinal SGK1/ENaC signaling and destroying gut *Lactobacillus*-maintained systemic type 1 interferon

**DOI:** 10.3389/fcimb.2026.1794494

**Published:** 2026-06-02

**Authors:** Bangtao Chen, Jing Yang, Fei Hao, Tingting Song

**Affiliations:** 1 Department of Dermatology, Chongqing University Three Gorges Hospital, School of Medicine, Chongqing University, Chongqing, China; 2 Department of Dermatology, Third Affiliated Hospital of Chongqing Medical University, Chongqing, China

**Keywords:** atopic dermatitis, ENaC, gut lactobacillus, high-salt-diet, SGK1, skin sodium, type 1 interferon

## Abstract

**Objective:**

Systemic type 1 interferon (IFN1), which is regulated by intestinal homeostasis, shows potential in inhibiting the immunoglobulin E (IgE) production and Th2 response *in vivo*. This study aimed to determine the effect of a high-salt diet (HSD) on systemic IFN1, gut microbiota and tight junction protein zonula occludens-1 (ZO-1), intestinal serum- and glucocorticoid-inducible kinase-1 (SGK1), and epithelial sodium ion channels (ENaCs) in atopic dermatitis (AD) mice.

**Methods:**

MC903 was applied topically to establish AD in wild-type C57BL/6 mice; in mice with IFN1 receptor-1 (*ifnar1*) knockout, *sgk1* conditional knockout (cKO) in intestinal epithelial cells (IECs), or stimulator of interferon genes (*sting*) cKO in dendritic cells (DCs); and in *BDCA2*-DTR mice with plasmacytoid DC (pDC) deletion.

**Results:**

HSD significantly promoted AD accompanied by higher levels of serum IgE, IL-4/IL-13, ileum SGK1, phosphorylated SGK1, βENaC and γENaC, and skin sodium, but lower levels of serum IFN1, ileum ZO-1, gut *Lactobacillus_murinus*, and *Lactobacillus_reuteri*. *Lactobacillus* gavage alleviated AD, reduced serum IgE and IL-4/IL-13, and increased serum IFN1 and ileum ZO-1 in AD mice on HSD, which were largely counteracted by *ifnar1* knockout, pDC deletion, or *sting* cKO in DCs, but not in IECs. cKO of *sgk1* in IECs alleviated AD, slightly reduced the serum IgE and IL-4/IL-13, and significantly reduced the ileum βENaC and γENaC and the skin sodium in mice on HSD without changing the gut microbiota, ileum ZO-1, and serum IFN1 levels.

**Conclusion:**

HSD promotes AD by partially impairing the systemic IFN1 production that is orchestrated by STING signaling in DCs by reducing the gut *Lactobacillus* and by partially increasing the sodium accumulation in skin lesions via driving the ileum SGK1 phosphorylation and βENaC and γENaC expression. Further research is necessary to assess the effect of *Lactobacillus* supplementation on clinical AD patients with an HSD habit.

## 1 Introduction

Atopic dermatitis (AD) is the most common chronic inflammatory dermatosis accompanied by chronic pruritus or acute itching episodes (Xie et al., 2023; Kim et al., 2024). Elevated Th2 cytokines in the serum or skin lesions and immunoglobulin E (IgE) in the serum are the typical pathophysiological characterization of AD; however, Th1 and Th17/Th22 cytokines are also increased at different stages of AD. The polarization of T helper (Th) cell subtypes and related Th cytokine production are synergistically regulated by multiple organs or systems, and the complex regulatory network also exists in IgE production (Schuler et al., 2023; Afshari et al., 2024; Guttman-Yassky et al., 2025). Therefore, emerging academic viewpoints and clinical observations recognize that AD is a systemic disease involving interactions among multiple systems and is not limited to skin manifestations.

More recently, the important regulation of stimulator of interferon genes (STING)/type 1 interferon (IFN1) signaling in non-viral and non-tumor diseases has been gradually revealed. For example, spinal STING/IFN1 signaling has been proven to confer protection against chronic itch in mice through inhibiting the phosphorylation of spinal extracellular signal-regulated kinase (Li et al., 2023). Both IFN1 signaling at steady-state and exogenous IFN1 can suppress ovalbumin (OVA)-specific IgE production and IgE-mediated systemic anaphylaxis in C57BL/6J mice (Chen B. et al., 2025). There were clinical studies attempting to treat AD with exogenous IFN1 30 years ago, but with inconsistent conclusions reached (MacKie, 1990; Michils et al., 1994; Kimata et al., 1995). It is noteworthy that the Th2 responses in non-AD diseases have also been uncovered to be suppressed by IFN1 signaling (Huber et al., 2010; Chen et al., 2020; Lin et al., 2020).

Emerging evidence demonstrates that systemic IFN1 homeostasis is precisely regulated by the gut microbiota in viral infections and tumorigenesis (Wirusanti et al., 2022). Disruption of the gut microbiota is considered one of the key mechanisms underlying AD onset (Mohammad et al., 2024). It is generally accepted that the gut microbiota is influenced by internal and external factors, and a high-salt diet (HSD) may shape specific types of gut microbiota in different diseases (Li et al., 2019; Wang et al., 2024). The sodium chloride in food is absorbed mainly through the epithelial sodium ion channels (ENaCs) in intestinal epithelial cells (IECs). ENaCs are hetero-multimeric complexes consisting of three homologous subunits (α, β, and γ). In renal tubules, the expression of ENaCs is controlled by serum and glucocorticoid inducible kinase-1 (SGK1) through inhibiting the E3 ligase activity of neural precursor cell-expressed developmentally downregulated 4-like (NEDD4L) by catalyzing NEDD4L phosphorylation (Debonneville et al., 2001; Liang et al., 2006; Lee et al., 2008; Al-Qusairi et al., 2016). However, the role of SGK1/NEDD4L signaling on intestinal ENaC abundance has not been determined. Recent clinical data suggest a close positive correlation between AD severity and HSD; however, there is a lack of animal experiments validating this and unveiling its possible mechanisms (Matthias et al., 2019; Chiang et al., 2024).

This study aimed to confirm the effect of HSD on the severity of AD in an MC903-induced murine AD model and to further investigate possible mechanisms by assessing the intestinal SGK1/NEDD4L/ENaC signaling and the gut microbiota-maintained systemic IFN1.

## 2 Materials and methods

### 2.1 Reagents

The following reagents were used: MC903 (no. HY-10001; MCE, Monmouth Junction, NJ, USA), diphtheria toxin (DT) (no. D0564; Sigma-Aldrich, St. Louis, MO, USA), mouse direct PCR kit for genotyping (no. B40015; Selleck, Houston, TX, USA), fecal genomic DNA extraction kit (no. DP328; TIANGEN, Beijing, China), QIAquick gel extraction kit (no. 28706X4; Qiagen, Hilden, Germany), ChamQ Blue Universal SYBR qPCR Master Mix (no. Q312-02; Vazyme, Nanjing, China), dual-luciferase reporter assay kit (no. DL101-01; Vazyme), enhanced chemiluminescence detection kit (no. WBKLS; Millipore, Darmstadt, Germany), and hematoxylin and eosin (HE) kit (no. DH0020; Leagene, Beijing, China). *Lactobacillus_ murinus* (no. Bio-03587) and *Lactobacillus_reuteri* (no. bio-53258) were purchased from biobw (Beijing, China). The enzyme-linked immunosorbent assay (ELISA) kits included mouse IL-4 (no. E-HSEL-M0002; Elabscience, Wuhan, China), mouse IL-13 (no. 0173M2; MEIMIAN, Jiangsu, China), and mouse total IgE (no. ab157718; Abcam, Cambridge, UK). The antibodies were anti-NEDD4L (no. A8085; ABclonal, Woburn, MA, USA), anti-phosphorylated NEDD4L (Ser448, no. AP0843; ABclonal), anti-GAPDH (no. A19056; Abclonal), anti-vinculin (no. AG3539; Beyotime, Shanghai, China), anti-SGK1 (no. 12103; CST, Danvers, MA, USA), phosphorylated SGK1 (Ser78, no. 5599; CST), anti-αENaC (no. ab272878; Abcam), anti-βENaC (no. A1765; Abclonal), anti-γENaC (no. A15097; Abclonal), and anti-zonula occludens-1 (ZO-1) (no. GB151981; Servicebio, Wuhan, China).

### 2.2 Mice

The animal experiments and procedures in this study were approved by the Ethics Committee of Chongqing University Three Gorges Hospital (no. SXYYWD2024-118). Furthermore, the experiments adhered to the guidelines on animal protection, Laboratory animal—Guidelines for the ethical review of animal welfare (GB/T 35892-2018, China). Chinese Cyagen Biosciences Inc. (Suzhou, Jiangsu Province, China) provided the wild-type (*wt*), *sgk1* flox (*f/f*), Cd11c^Cre^, and Vil1^Cre^ C57BL/6 mice. *sting* flox (*f*/*f*) and *Ifnar1* knockout (*Ifnar1*^KO^) C57BL/6 mice were obtained from Shanghai Model Organisms Center, Inc. (Shanghai, China). *BDCA2*-DTR mice were a kind gift from Professor Rui He (Fudan University). Mice with conditional knockout (cKO) of *sgk1* in IECs were obtained by mating *sgk1*(*f*/*f*) mice with Vil1^Cre^ mice. cKO of *sting* in dendritic cells (DCs) and IECs were obtained by mating *sting*(*f*/*f*) mice with Cd11c^Cre^ and Vil1^Cre^ mice, respectively. To delete plasmacytoid DCs (pDCs) *in vivo*, BDCA2-DTR mice were given DT (0.01 mg/kg) by intraperitoneal injection three times weekly during HSD according to literature reports (Swiecki and Colonna, 2010; Mandl et al., 2015).

All mice were bred and housed in ventilated cages on the same housing unit (room temperature, 25 ± 2°C; humidity, 50% ± 5%; light/dark, 12 h/12 h) in specified pathogen-free room.

### 2.3 AD mouse model and interventions

Of MC903 (45 μM), 20 μl/cm^2^ was topically applied to the exposed nape of the mice for 10–14 consecutive days to induce AD-like dermatitis. At 2 weeks prior to and during MC903 application, mice were fed with a normal-salt diet (NSD) or a HSD. Approximately 3% of pentobarbital sodium (5 μl/g) was intraperitoneally injected to induce anesthesia, and related samples, including serum, skin lesions, and feces from the ileum and ileum tissues, were collected.

### 2.4 Bacterial supplementation

C57BL/6 mice at 8 weeks old were orally administered live *Lactobacillus* (*murinus*/*reuteri* = 1:1) at a dose of 1 × 10^9^ colony-forming units (CFU) in 200 µl anaerobic saline daily starting from the HSD. Control groups received an equal volume of anaerobic saline.

### 2.5 Dual-luciferase reporter gene assay

The total serum IFN1 level was indirectly evaluated by assessing the interferon-stimulated response element (ISRE) activity induced by serum samples on HEK293T cells stably expressing IFN1 receptors, as determined with a dual-luciferase reporter gene assay, according to the methods described in our previous study (Chen B. et al., 2025).

### 2.6 Skin sodium determination

Sodium in fresh skin biopsies was analyzed using inductively coupled plasma optical emission spectrometry (ICP-OES) as described in Soll et al. (2024).

### 2.7 Enzyme-linked immunosorbent assay

The serum total IgE, IL-4, and IL-13 levels were determined using the corresponding ELISA kits following the manufacturers’ instructions.

### 2.8 16S rDNA sequencing

Following sacrifice, fresh fecal samples from the ileum were collected for 16S rDNA sequencing using bacterial universal primers targeting the V3–V4 regions. Sequencing and bioinformatics analyses were performed as previously described (Chen B. et al., 2025). Briefly, paired-end raw reads with overlap were merged to tags, and tags were clustered to operational taxonomic units (OTUs) at 97% sequence similarity using UPARSE (version 7.1; http://drive5.com/uparse/). Alpha diversity indices (Shannon and Simpson), evenness indices (simpsoneven and shannoneven), and richness estimators [e.g., abundance-based coverage estimator (ACE), observed species (*S*_obs_), and Chao1] at the OTU level were calculated using Mothur v.1.30.2. Beta diversity assessment using principal coordinate analysis (PCoA) was conducted using R package and analysis of similarities (ANOSIM) using R-vegan. Statistically significant differences in the relative abundance of taxa at the species level were determined with the Wilcoxon rank-sum test. The raw reads were deposited into the NCBI Sequence Read Archive database (accession no. PRJNA1452548).

### 2.9 Western blotting

The details for Western blotting and protein visualization have been previously described (Chen et al., 2023). The primary antibodies used included anti-SGK1, anti-NEDD4L, anti-phosph-SGK1, anti-phosph-NEDD4L, anti-αENaC, anti-βENaC, anti-γENaC, anti-vinculin, and anti-GAPDH.

### 2.10 Histological stain

Fresh skin lesions were cut into 0.5 × 0.5-cm^2^ sections, fixed in 4% paraformaldehyde for 24 h, and embedded in paraffin. Deparaffinized and rehydrated slices were stained with HE for measuring the epidermal thickness and observing the inflammatory infiltration. Immunofluorescence was performed to assess the expression of the tight junction protein ZO-1 in the ileum with the anti-ZO-1 mouse monoclonal antibody (1:500) and specific antigen retrieval conditions (EDTA 8.0, water bath at 90°C for 30 min).

### 2.11 Statistical analysis

Continuous variables were expressed as the mean (*M*) ± standard deviation (SD). GraphPad Prism® 5.0 software package (San Diego, CA, USA) was used for statistical analysis. One-way ANOVA and Bonferroni’s or Dunnett’s multiple comparison post-tests were used in the analysis of different groups. A two-sided *p*-value <0.05 was considered statistically significant.

## 3 Results

### 3.1 HSD promoted MC903-induced AD

**Figure 1:**
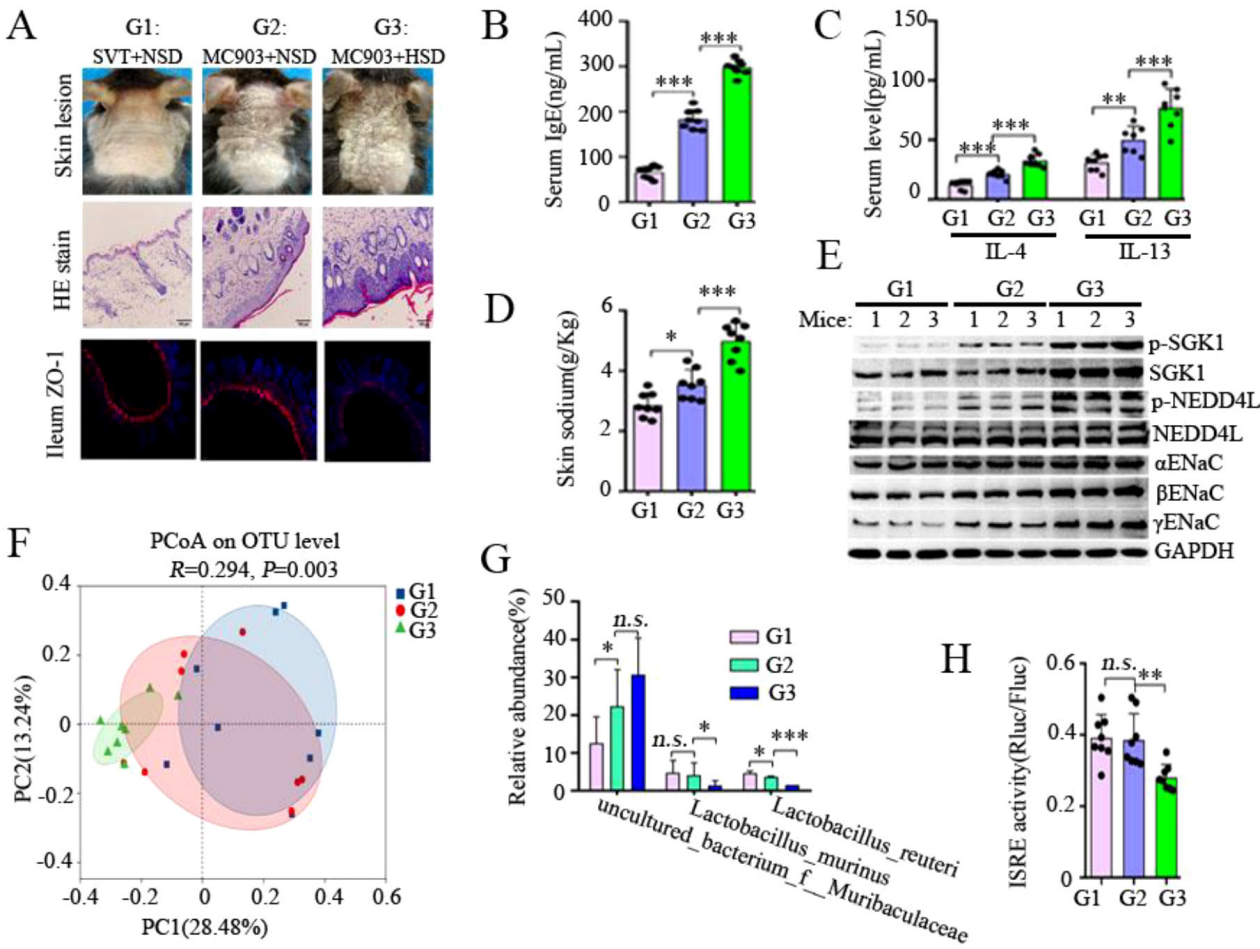
Effect of a high-salt diet (HSD) on atopic dermatitis (AD) severity, skin sodium, gut homeostasis, and type 1 interferon (IFN1) level. MC903 (45 μM, 20 μl/cm^2^) or an equivalent SVT was topically applied to the exposed nape of 8- to 10-week-old mice (eight male mice per group) for 12 consecutive days. Mice received either normal-salt diet (NSD) or HSD for 2 weeks prior to and during MC903 application. The mice were then anesthetized and related samples were obtained. Skin lesions, hematoxylin–eosin (HE) staining of the lesions, and the ileum zonula occludens-1 (ZO-1) protein expression **(A)** (bar represents 100 μm), serum IgE **(B)**, serum IL-4 and IL-13 **(C)**, skin sodium **(D)**, related proteins in the ileum **(E)**, β-diversity based on principal coordinate analysis **(F)**, the relative abundance of the main differential gut microbiota at the species level **(G)**, and the total serum IFN1 **(H)** levels are shown. Statistics: one-way ANOVA + Bonferonni’s tests. n.s., *p* > 0.05; *p < 0.05, **p < 0.01, ***p < 0.001.

Topical application of MC903 for 12 consecutive days in adult *wt* C57BL/6 mice on an NSD caused obvious megascopic skin lesions, inflammatory cell infiltration in the dermis, epidermis thickening (**Figure 1A**), and a significant increase in the serum levels of total IgE (**Figure 1B**), IL-4, and IL-13 (**Figure 1C**) and skin sodium (**Figure 1D**) compared with the corresponding solvent (SVT) treatment mice. In the condition of MC903 challenge, more severe skin lesions accompanied by more inflammatory infiltration and thicker epidermis, as well as higher levels of serum total IgE, IL-4, and IL-13 and skin sodium (**Figures 1A–D**), were observed in mice on an HSD than those on an NSD, which clearly demonstrates the promoting effect of HSD on AD severity in mice.

### 3.2 HSD affected systemic IFN1 and gut homeostasis in AD mice

In mice on NSD, the levels of ileum SGK1 and NEDD4L phosphorylation and ileum γENaC, but not βENaC and αENaC, were slightly increased upon MC903 challenge; however, these and ileum βENaC were all remarkably increased in the HSD+MC903 group than the NSD+MC903 group, and ileum SGK1 was also significantly upregulated in the HSD+MC903 group (**Figure 1E**). Immunofluorescence detection showed similar expression levels in ileum ZO-1 between the NSD+SVT and NSD+MC903 groups, but lower expression in the HSD+MC903 group, indicating that HSD, but not MC903, increases intestinal permeability (**Figure 1A**; **Supplementary Figure 1A**). The gut microbiota was further evaluated. Comparable index values for ACE, *S*_obs_, Chao1, Shannon, Simpson, shannoneven, and simpsoneven were found between the NSD+SVT and NSD+MC903 groups, or between the NSD+MC903 and HSD+MC903 groups, demonstrating no significant differences in the fecal bacterial community richness, α-diversity, or evenness between the indicated two groups (**Supplementary Figure 2A**). Unlike the NSD+SVT and NSD+MC903 groups, the samples in the HSD+MC903 group were highly concentrated in a different cluster on PCoA at the OTU level (*R* = 0.294, *p* = 0.003) (**Figure 1F**). At the species level, the relative abundance rates of *uncultured_bacterium_f:Muribaculaceae* (*p* < 0.05) and *Lactobacillus_reuteri* (*p* < 0.05) in the NSD+MC903 group were significantly higher and lower than that in the NSD+SVT group, respectively. Compared with the NSD+MC903 group, *Lactobacillus_murinus* (*p* < 0.05) and *Lactobacillus_reuteri* (*p* < 0.001) were significantly lower and *uncultured_bacterium_f:Muribaculaceae* slightly higher (*p* > 0.05) in the HSD+MC903 group (**Figure 1G**). Finally, the total serum IFN1 level was indirectly measured, and the results showed that it remained similar between the NSD+SVT and NSD+MC903 groups, but was much lower in the HSD+MC903 group (**Figure 1H**). These data suggest that the gut homeostasis and systemic IFN1 level are impaired by HSD in the MC903-induced murine AD model, and the upregulation in the ileum βENaC and γENaC orchestrated by SGK1/NEDD4L signaling may promote gut sodium absorption, leading to an increase in the sodium accumulation in skin lesions.

### 3.3 *Lactobacillus* supplementation improving AD was partially dependent on IFN1

**Figure 2:**
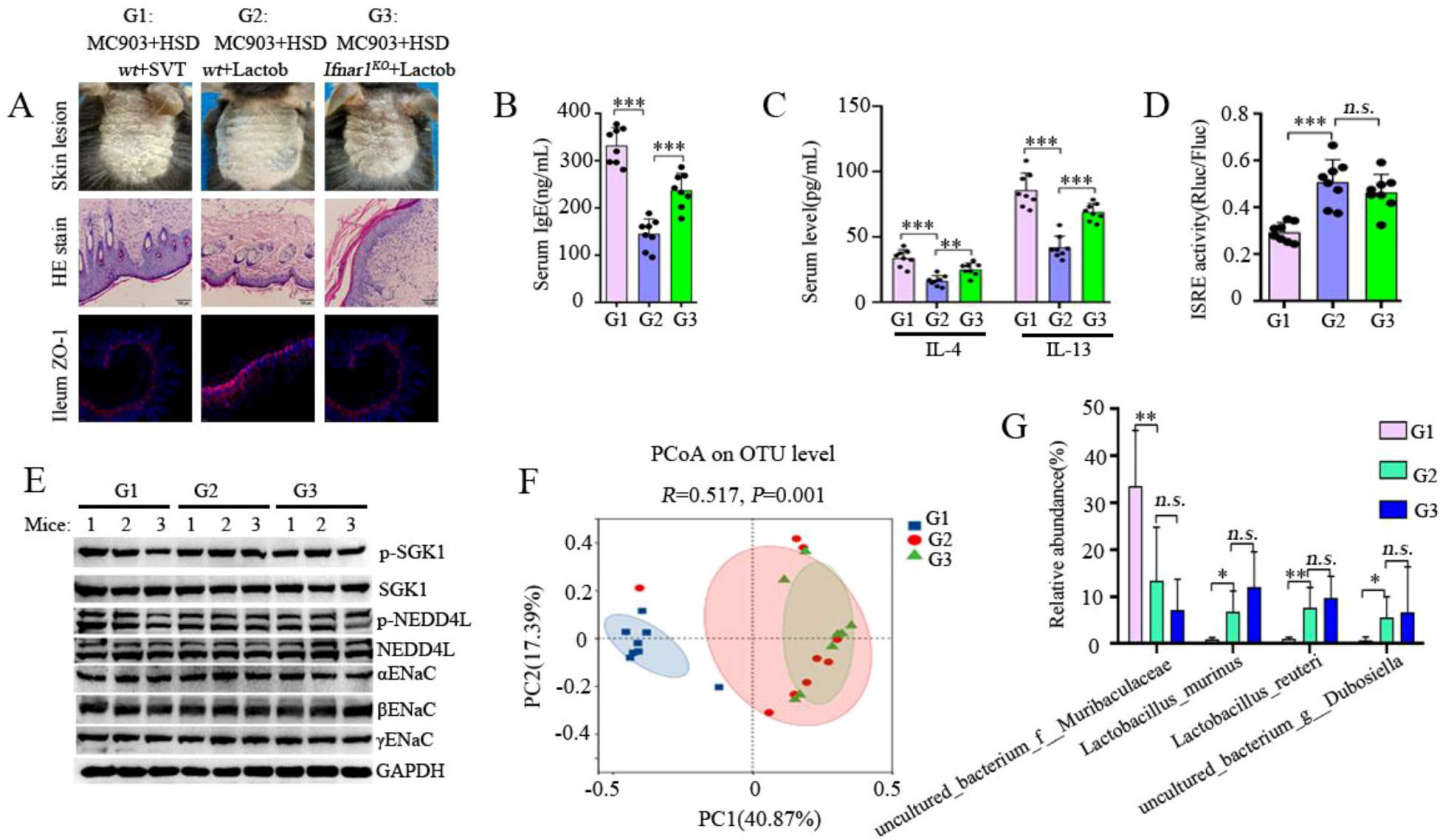
Effect of *Lactobacillus* supplementation on atopic dermatitis (AD) and its correlation with gut homeostasis and the type 1 interferon (IFN1) level in mice receiving a high-salt diet (HSD). MC903 (45 μM, 20 μl/cm^2^) was topically applied to the exposed nape of wild-type (*wt*) or *Ifnar1* knockout (*Ifnar1*^KO^) mice (8–10 weeks old, eight male mice per group) for 12 consecutive days. Mice synchronously received HSD and SVT or *Lactobacillus* (*Lactob*) supplementation for 2 weeks prior to and during MC903 application. The mice were then anesthetized and related samples were obtained. Skin lesions, hematoxylin–eosin (HE) staining of the lesions, and ileum zonula occludens-1 (ZO-1) protein expression **(A)** (bar represents 100 μm), serum IgE **(B)**, serum IL-4 and IL-13 **(C)**, total serum IFN1 level **(D)**, related proteins in the ileum **(E)**, β-diversity based on principal coordinate analysis **(F)**, and the relative abundance of the main differential gut microbiota at species level **(G)** are shown. Statistics: One-way ANOVA + Bonferroni’s tests. n.s., *p* > 0.05; *p < 0.05, **p < 0.01, ***p < 0.001.

To confirm the relationships among gut homeostasis, systemic IFN1 level, and AD degree, *wt* or *Ifnar1*^KO^ mice that received HSD+MC903 were orally administered *Lactobacillus*. **Figure 2A** shows that the epidermal thickness in *wt* mice was alleviated by *Lactobacillus* administration, accompanied by reduced serum IgE (**Figure 2B**) and IL-4 and IL-13(**Figure 2C**), but increased serum IFN1 level (**Figure 2D**) and ileum ZO-1 expression (**Figure 2A**; **Supplementary Figure 1B**). Among mice that received HSD+MC903+*Lactobacillus*, compared with the *wt*, the epidermal thickness and the serum IgE, IL-4, and IL-13 in *Ifnar1*^KO^ mice were significantly increased and the ileum ZO-1 expression was also downregulated, but did not reach the levels observed in *wt* mice that received HSD+MC903. Noticeably, the levels of ileum αENaC, βENaC, γENaC, and phosphorylated SGK1/NEDD4L were all unchanged among the three groups (**Figure 2E**). Lower index values for ACE (*p* < 0.01), *S*_obs_ (*p* < 0.01), and Chao1 (*p* < 0.01) were found in *wt* mice that received HSD+MC903+*Lactobacillus* compared with those that received HSD+MC903, but remained similar between *wt* and *Ifnar1*^KO^ mice that received HSD+MC903+*Lactobacillus* (**Supplementary Figure 2B**). Unlike the other two groups, the samples in *wt* mice that received HSD+MC903 were highly concentrated in a different cluster on PCoA at the OTU level (*R* = 0.517, *p* = 0.001) (**Figure 2F**). At the species level (**Figure 2G**), the relative abundance rates of *Lactobacillus_murinus* (*p* < 0.05), *Lactobacillus_reuteri* (*p* < 0.01), and *uncultured_bacterium_g:Dubosiella* (*p* < 0.05) were significantly higher, while that of *uncultured_bacterium_f:Muribaculaceae* (*p* < 0.01) was remarkably lower in *wt* mice that received HSD+MC903+*Lactobacillus* than in mice receiving HSD+MC903. No significant differences in the relative abundance of any bacteria between *wt* and *Ifnar1*^KO^ mice that received HSD+MC903+*Lactobacillus* were observed. These data suggest that *Lactobacillus* supplementation improving AD under HSD is partially dependent on the systemic IFN1 level.

### 3.4 STING signaling in DCs was required for *Lactobacillus* improving AD

**Figure 3:**
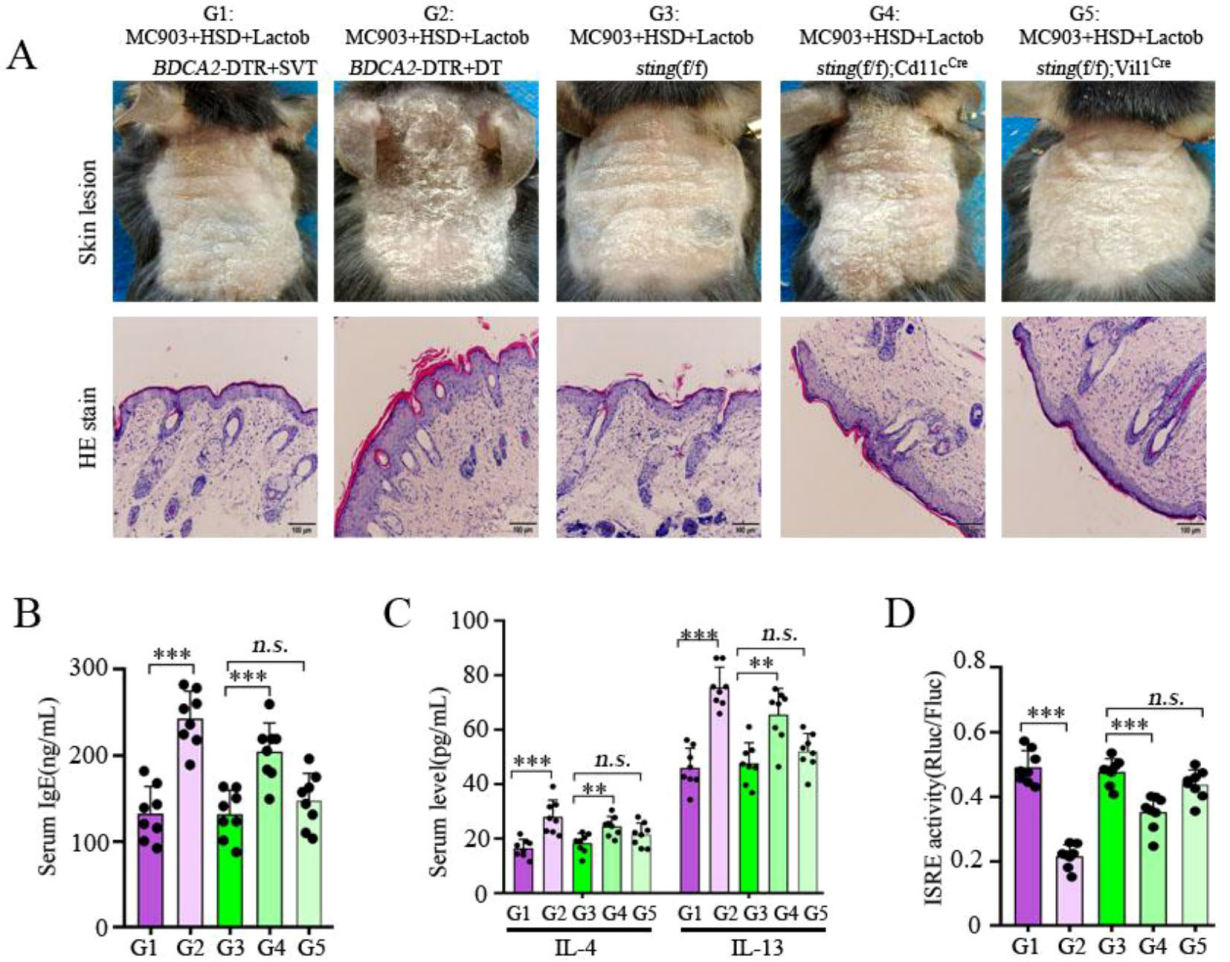
Role of plasmacytoid dendritic cells (pDCs) or stimulator of interferon genes (STING) signaling in *Lactobacillus*-mediated atopic dermatitis (AD) alleviation in mice receiving a high-salt diet (HSD). MC903 (45 μM, 20 μl/cm^2^) was topically applied to the exposed nape of *BDCA2*-DTR mice, *BDCA2*-DTR mice with diphtheria toxin (DT) intraperitoneal injection, and mice with positive *sting*(*f/f*), *sting*(*f/f*);Cd11c^Cre^, or *sting*(*f/f*);Vil1^Cre^ (8–10 weeks old, eight male mice per group) for 12 consecutive days. All mice synchronously received HSD and *Lactobacillus* (*Lactob*) supplementation for 2 weeks prior to and during MC903 application. The mice were then anesthetized and related samples were obtained. Skin lesions and hematoxylin–eosin (HE) staining of the lesions **(A)** (bar represents 100 μm), serum IgE **(B)**, serum IL-4 and IL-13 **(C)**, and the total serum type 1 interferon (IFN1) level **(D)** are shown. Statistics: One-way ANOVA + Bonferroni’s tests. n.s., *p* > 0.05; **p < 0.01, ***p < 0.001.

To determine whether *Lactobacillus*-mediated systemic IFN1 maintenance is dependent on the existence of pDCs and STING signaling in DCs or IECs in mice that received HSD+MC903+*Lactobacillus*, DT was first intraperitoneally injected in *BDCA2*-DTR mice to delete pDCs. The epidermal thickness was aggravated, accompanied by increased serum IgE and IL-4/IL-13 levels, but a decreased serum IFN1 upon pDC deletion (**Figures 3A–D**). Compared with the *sting*(*f/f*) mice, cKO of *sting* in DCs also significantly upregulated the epidermal thickness and the serum IgE and IL-4/IL-13, but reduced the serum IFN1; however, the change degrees in these indicators were not as significant as those observed under the condition of pDC deletion (**Figures 3A–D**). In addition, cKO of *sting* in IECs only slightly reduced the serum IFN1 and increased the serum IgE and IL-4/IL-13, but showed no effect on epidermal thickness (**Figures 3A–D**). These data suggest that *Lactobacillus* maintaining the systemic IFN1 level in AD mice on HSD is pDC-dependent and that STING signaling in DCs contributes the vast majority.

### 3.5 Ablation of *sgk1* in IECs alleviated HSD/MC903-induced AD by reducing skin sodium without affecting IFN1

**Figure 4:**
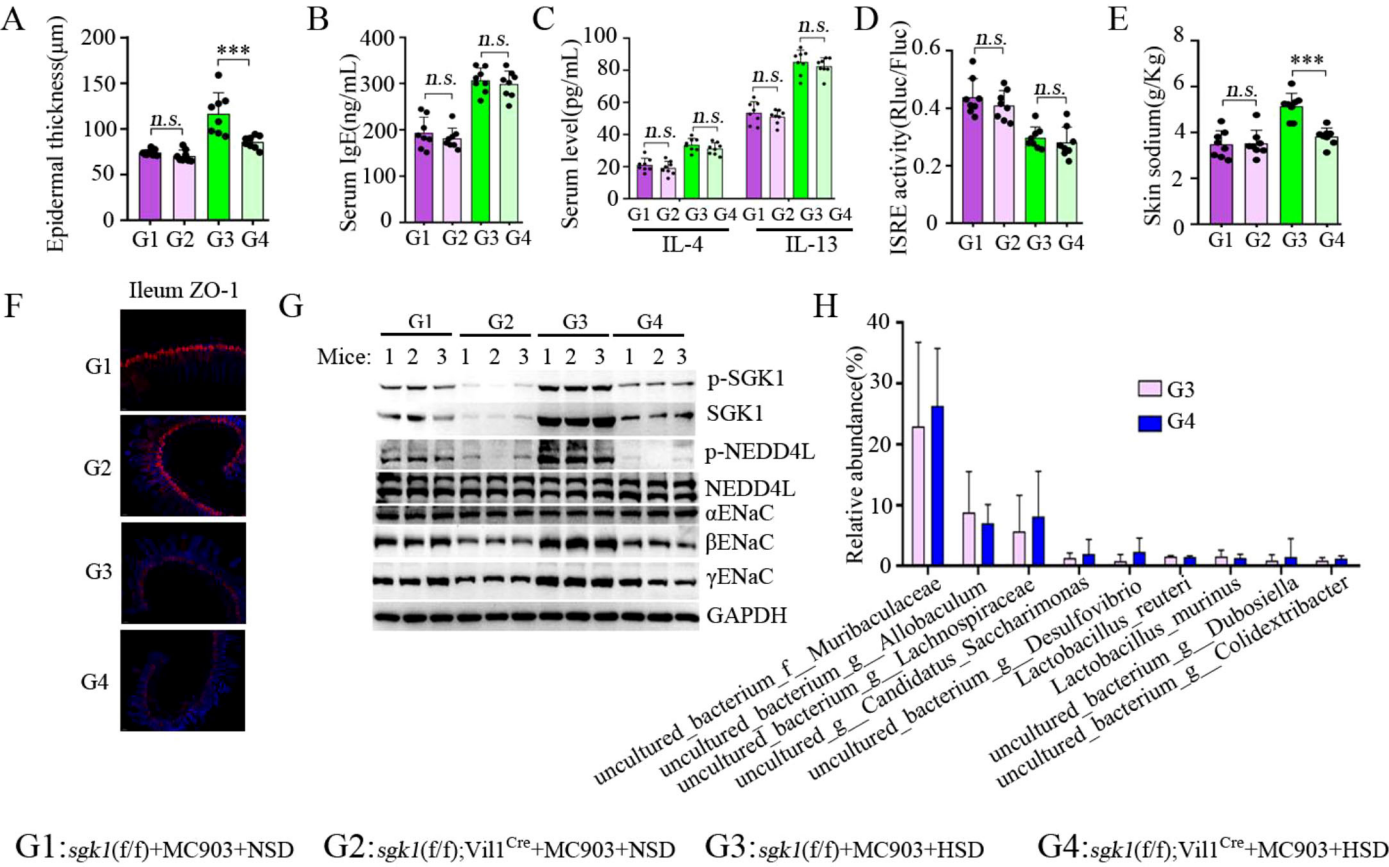
Role of intestinal epithelial cell (IEC)-specific SGK1 signaling in atopic dermatitis (AD) and its correlation with the gut homeostasis and type 1 interferon (IFN1) level in mice receiving a high-salt diet (HSD) or a normal-salt diet (NSD). MC903 (45 μM, 20 μl/cm^2^) was topically applied to the exposed nape of mice with *sgk1*(*f/f*) or *sgk1*(*f/f*);Vil1^Cre^ (8–10 weeks old, eight male mice per group) for 12 consecutive days. Mice received HSD for 2 weeks prior to and during MC903 application. The mice were then anesthetized and related samples were obtained. Epidermal thickness **(A)**, serum IgE **(B)**, serum IL-4 and IL-13 **(C)**, and total serum IFN1 level **(D)**, skin sodium **(E)**, ileum zonula occludens-1 (ZO-1) protein expression **(F)**, related proteins in the ileum **(G)**, and the relative abundance of main the differential gut microbiota at the species level **(H)** are shown. Statistics: One-way ANOVA + Bonferroni’s tests. n.s., *p* > 0.05; ***p < 0.001.

Considering the possible pivotal role of intestinal SGK1 in gut sodium absorption by regulating intestinal ENaC degradation, mice with cKO of *sgk1* in IECs [*sgk1*(*f/f*);Vil1^Cre^] were further applied. Similar epidermal thickness (**Figure 4A**), comparable levels of serum IgE (**Figure 4B**), IL-4 and IL-13 (**Figure 4C**), and IFN1 (**Figure 4D**), skin sodium (**Figure 4E**), and ileum ZO-1 expression (**Figure 4F**; **Supplementary Figure 1C**) were observed between *sgk1*(*f/f*);Vil1^Cre^ mice and *sgk1*(*f/f*) mice that received NSD+MC903, with a slight reduction in the ileum βENaC and γENaC in the former (**Figure 4G**). Compared with *sgk1*(*f/f*) mice that received HSD+MC903, the epidermal thickness, the ileum βENaC and γENaC, and the skin sodium were all reduced (all *p* < 0.05), but the levels of serum IgE, IL-4, IL-13, and IFN1 and the ileum ZO-1 expression were not changed in *sgk1*(*f/f*);Vil1^Cre^ mice that received HSD+MC903 (all *p* > 0.05). Analysis of the gut microbiota showed that the fecal bacterial community richness, α-diversity, evenness (**Supplementary Figure 2C**), and β-diversity (**Supplementary Figure 3**), as well as the composition and abundance of gut bacteria, were similar between *sgk1*(*f/f*);Vil1^Cre^ mice and *sgk1*(*f/f*) mice that received HSD+MC903 (**Figure 4H**). Finally, similar α-diversity (**Supplementary Figure 2D**), β-diversity, and taxon abundance of the gut bacteria (**Supplementary Figure 4**) were also observed between *sgk1*(*f/f*);Vil1^Cre^ mice and *sgk1*(*f/f*) mice on NSD without MC903 exposure. These data imply that intestinal SGK1 deletion partially improves AD by reducing the intestinal ENaC upregulation and skin sodium accumulation only in mice on HSD, which is independent of the regulation of IgE production, Th2 response, gut microbiota, and systemic IFN1.

## 4 Discussion

HSD is an extremely common lifestyle issue, and it is of great translational significance to elucidate the exact effects of HSD on AD. A cross-sectional study involving 215,832 adult individuals with 10,839 AD cases showed that the occurrence and severity of AD are positively associated with a 1-g increase in the estimated 24-h urine sodium excretion. A recall questionnaire survey also demonstrated that HSD increases the risk of current AD (Chiang et al., 2024). Moreover, the skin sodium content was found to increase in skin lesions from patients with AD, but remain similar in non-lesional skin from patients with AD and the skin from healthy volunteers (Matthias et al., 2019). In this study based on an MC903-induced AD murine model, we confirmed that skin sodium is upregulated in AD-like lesions and that HSD aggravates AD severity, accompanied by higher levels of sodium in the skin lesions and IgE and IL-4/IL-13 in the serum. Similar to the phenomena we observed, Matthias et al. provided proof that sodium chloride promotes Th2 inflammation, the differentiation and polarization of Th2 cells from naive T-cell precursors, through osmosensitive transcription factor nuclear factor of activated T-cell 5 (NFAT5) and the increased kinase SGK1 in memory Th cells (Matthias et al., 2019). Liu et al. also found higher levels of serum IgE and IL-4 in a mouse model of oral OVA allergy maintained on a HSD (Liu et al., 2021). Musiol et al. demonstrated that HSD-fed mice display an aggravated mite-induced allergic airway inflammation characterized by increased levels of total serum IgE and Th2 and Th17 inflammation in the respiratory tract (Musiol et al., 2024).

In addition to the T-cell response, the gut microbiota is the most important target for HSD, which is also disrupted in AD mice on NSD. Higher abundance rates of the genera *Limosilactobacillus* and *Parasutterella*, whilebut lower *Bifidobacterium*, *Helicobacter*, *Mucispirillum*, and *Holdemania* abundance, were observed in topical MC903-treated than solvent-treated C57BL/6J mice (Yeom et al., 2024). Hou et al. found that the relative abundance of *Bifidobacterium*, *Lactobacillus*, *Lachnoclostridium*, *Turicibacter*, and *Enterorhabdus* decreased, while *Roseburia*, *Gordonibacter*, *Odoribacter*, *Bacteroides*, and *Anaerotruncus* increased in an MC903-induced AD murine model (Hou et al., 2020). Partially similar to these findings, a slight increase in the abundance of *uncultured_bacterium_f:Muribaculaceae* and a slight decrease in *Lactobacillus_reuteri* in AD mice on NSD were found in our study. Moreover, we displayed new evidence that AD mice on HSD show a slight increase in *uncultured_bacterium_f:Muribaculaceae*, but a significant decrease in *Lactobacillus_murinus* and *Lactobacillus_reuteri*, compared with AD mice on NSD. The downregulation effect of HSD on *Lactobacillus* is also observed in non-AD diseases. For example, HSD depletes gut *L. murinus* and thus increases the blood pressure in FVB/N mice and reduces the intestinal survival of *Lactobacillus* spp., accompanied by *Lactobacillus*-produced intestinal indole-3-lactic acid in healthy humans (Wilck et al., 2017; Jama and Marques, 2020). It also causes a substantial reduction in gut Lactobacillaceae and the serum level of 5-hydroxyindole acetic acid in mice with experimental autoimmune prostatitis (Chen J. et al., 2025).

The pathway through which HSD-mediated dysbacteriosis exacerbates AD is a key topic that we are focusing on. The IFN1 response regulated by the gut microbiota has gradually been recognized in viral and tumoral diseases, with our previous study also uncovering the gut microbiota/IFN1 axis in IgE-mediated allergic diseases (Chen B. et al., 2025). Non-clinical experiments have revealed the suppressive effect of IFN1 on human memory Th2 inflammation (Huber et al., 2010; Chen et al., 2020; Lin et al., 2020). However, clinical data indicate that subcutaneous administration of IFN1 for 6–12 weeks is not always effective in alleviating AD symptoms and the serum IgE level (MacKie, 1990; Michils et al., 1994; Kimata et al., 1995). It is speculated that this is largely attributed to the high heterogeneity of clinical AD. In this study, we demonstrated several lines of evidence unveiling the mystery of HSD promoting AD by destroying the *Lactobacillus*-maintained systemic IFN1. Firstly, HSD, and not NSD, remarkably downregulated the gut *Lactobacillus* abundance and the serum IFN1 level in AD mice. Secondly, *Lactobacillus* supplementation upregulated the serum IFN1 level and alleviated the AD degree and serum IgE and IL-4/IL-13 in AD mice on HSD, which was largely, although not entirely, blocked by *ifnar1* knockout. Thirdly, pDC deletion or cKO of *sting* in DCs, and not in IECs, significantly reduced the serum IFN1 and thus aggravated the AD degree and increased the serum IgE and IL-4/IL-13 in AD mice on HSD. These data collectively demonstrate that *Lactobacillus*-maintained systemic IFN1 suppresses AD in the condition of HSD via activating the STING signaling in pDCs, which also supports knowledge that pDCs are the main producers of systemic IFN1 (Monaghan et al., 2023; Ngo et al., 2024). In addition, there may be non-IFN1 factors playing roles in the inhibition of AD by *Lactobacillus* as the increased gut *uncultured_bacterium_f:Muribaculaceae* in AD mice on HSD was also reduced by *Lactobacillus* supplementation; however, the role of gut bacteria in AD development is still unknown. The evidence we provided further corroborates previous findings, indicating that supplementation with *Lactobacillus* offers benefits for patients with AD and mice with AD-like symptoms (Sun et al., 2025; Rønnstad et al., 2026).

Intestinal sodium absorption and the redistribution of sodium are inevitable pathophysiological processes following HSD challenge. It is well acknowledged that the sodium in food can be absorbed throughout the intestine, but mainly occurs in the small intestine, where it is actively transported in the jejunum and passively transported in the ileum with the sodium pump. Ileum ENaC abundance is negatively regulated by the E3 ligase activity of NEDD4L, but the activity is negatively regulated by phosphorylated SGK1. Palmada et al. provided proof that SGK1 is co-localized with NEDD4L in villus enterocytes from human small intestine by immunohistochemistry (Palmada et al., 2004). Aguiar et al. demonstrated that colon *sgk1* mRNA at 90 min is slightly upregulated in C57BL/6J mice that received a 50% sodium chloride solution by gavage, but which is significantly reduced after 120 h or 3 weeks of HSD (Aguiar et al., 2018). In an *in vitro* experiment, Zhang et al. showed that sodium chloride activates macrophages by increasing the SGK1 expression and phosphorylation (Zhang et al., 2024). In this study, slight increases in the ileum SGK1 and NEDD4L phosphorylation, as well as in γENaC expression, were found in AD mice on NSD, whereas remarkable increases in the ileum SGK1 expression, SGK1 and NEDD4L phosphorylation, and βENaC and γENaC expression were found in AD mice on HSD. The increased ileum ENaC expression due to SGK1-mediated NEDD4L phosphorylation may contribute to skin sodium accumulation in AD mice on NSD or HSD (**Figure 1**). We also found that the ileum SGK1 and NEDD4L phosphorylation and the ENaC expression are not influenced by *Lactobacillus* supplementation in wild-type or *ifnar1* knockout mice on HSD, which implies that the intestinal SGK1/NEDD4L signaling and the ENaC expression are unrelated to the *Lactobacillus*-maintained systemic IFN1 level in this setting. Intestinal SGK1 may become a potential target for the treatment of AD. In the current study, cKO of *sgk1* in IECs indeed reduced the ileum βENaC and γENaC expression by inhibiting the NEDD4L phosphorylation in AD mice on NSD or HSD; however, the AD degree and skin sodium were alleviated only in mice on HSD, which is not accompanied by impairments in the intestinal homeostasis and the systemic IFN1 level. These data demonstrate that intestinal SGK1 signaling promotes AD only in the setting of HSD via facilitating skin sodium accumulation without influencing the gut microbiota and the systemic IFN1 level.

Finally, the role of alterations in the gut permeability in AD may also provide important clues for clarifying the mechanism by which HSD exacerbates AD. Yeom et al. showed increased gut permeability in AD mice on NSD (Yeom et al., 2024). However, we found increased gut permeability only in AD mice on HSD rather than NSD, and it could be alleviated by *Lactobacillus* supplementation in an IFN1-dependent manner. Consistent with this finding, Hwang et al. also found that IFNβ could mitigate alcohol-induced gut permeability (Hwang et al., 2024). Moreover, the new evidence provided in this study suggests that the gut permeability is not related to the changes in the intestinal SGK1/NEDD4L signaling and the ENaC expression in AD mice on NSD or HSD.

## 5 Limitations

Several limitations exist in this study. Firstly, no validation experiments were performed to determine the effects of HSD on AD development, systemic IFN1, gut microbiota, and intestinal SGK1/NEDD4L/ENaC signaling based on specimens from clinical AD patients. Secondly, if the effects of HSD, *Lactobacillus*, or IFN1 on the SGK1/NEDD4L/ENaC signaling in IECs can be directly verified in *in vitro* experiments, the conclusions will be more convincing. Thirdly, whether the observed experimental phenomena also exist in female mice is not verified. Fourthly, it is still unclear which IFN1 subtype plays a major role in AD. Lastly, evaluating the homeostasis of other intestinal parts aside from the ileum will provide a more comprehensive understanding of the mechanism by which HSD affects AD.

## 6 Conclusions

HSD promotes AD by partially impairing the systemic IFN1 production orchestrated by STING signaling in DCs through reducing the gut *Lactobacillus* and partially increasing the sodium accumulation in skin lesions via driving the ileum SGK1 phosphorylation and the βENaC and γENaC expression.

## Data Availability

The original contributions presented in the study are included in the article/**Supplementary Material**. Further inquiries can be directed to the corresponding author.
