# Supplementary material for: High-salt diet promotes atopic dermatitis by partially enhancing intestinal SGK1/ENaC signaling and destroying gut Lactobacillus-maintained systemic type 1 interferon
Source: Front Cell Infect Microbiol. 2026 Jun 2;16:1794494. doi: 10.3389/fcimb.2026.1794494 (PMC13268906; doi:10.3389/fcimb.2026.1794494)
Supplement: Supplementary file 1 [file SupplementaryFile1.docx]

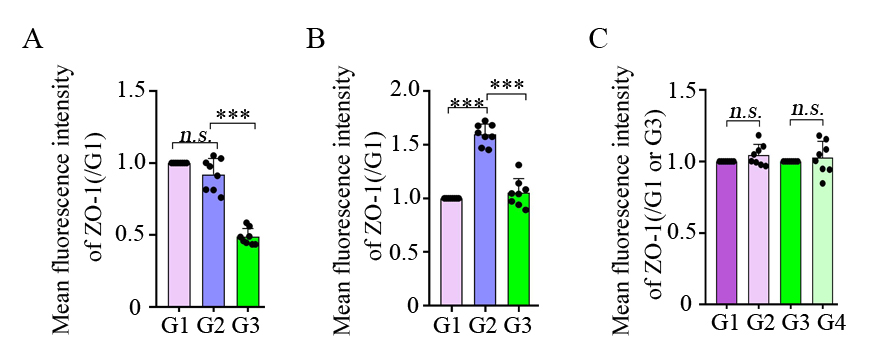


Supplementary Figure 1 Analysis of mean fluorescence intensity of ZO-1in Figure 1A(A), Figure 2A(B),and Figure 4F(C). Statistics: One-way ANOVA+ Bonferroni’s tests, n.s.*P* > 0.05, *** *P* < 0.001.


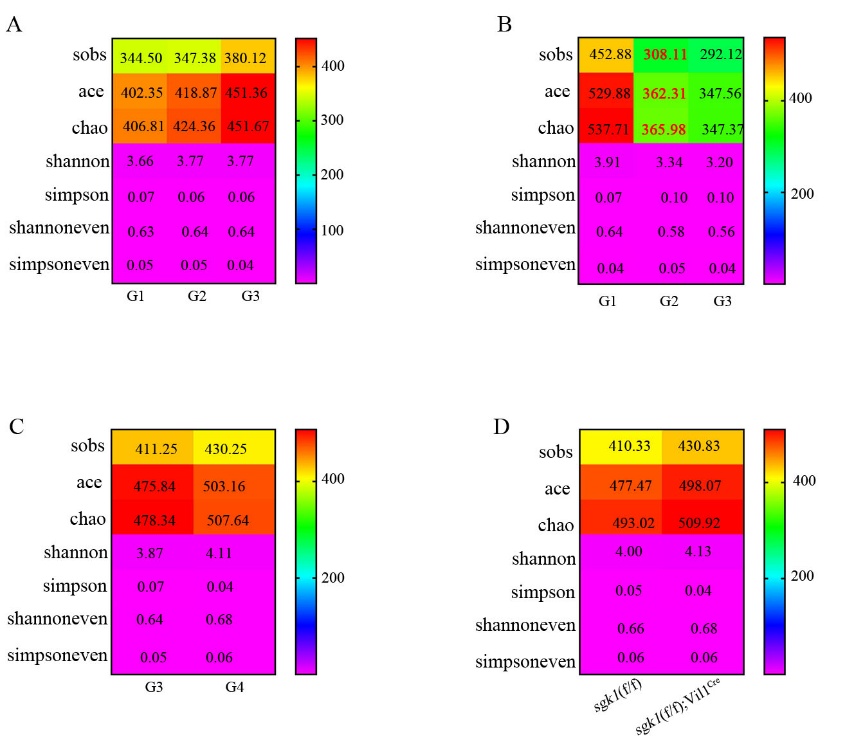


Supplementary Figure 2 Analysis of gut bacterial community richness, α-diversity, or evenness among groups. (A) the results among the three groups in Figure 1, (B) the results among the three groups in Figure 2, (C) the results between the two groups (G3 vs. G4) in Figure 4, (D) the results between the two groups [*sgk1*(f/f) vs. *sgk1*(f/f);Vil1^Cre^] are shown.


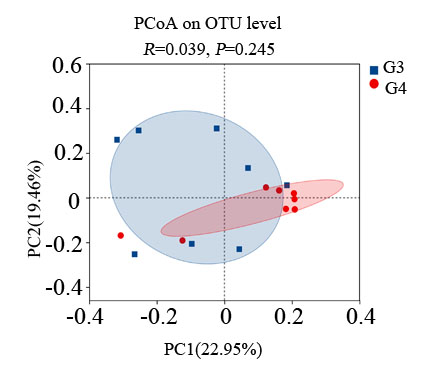


Supplementary Figure 3 Analysis of gut bacterial β-diversity based on principal coordinate analysis between group 3 and group 4 in figure 4.


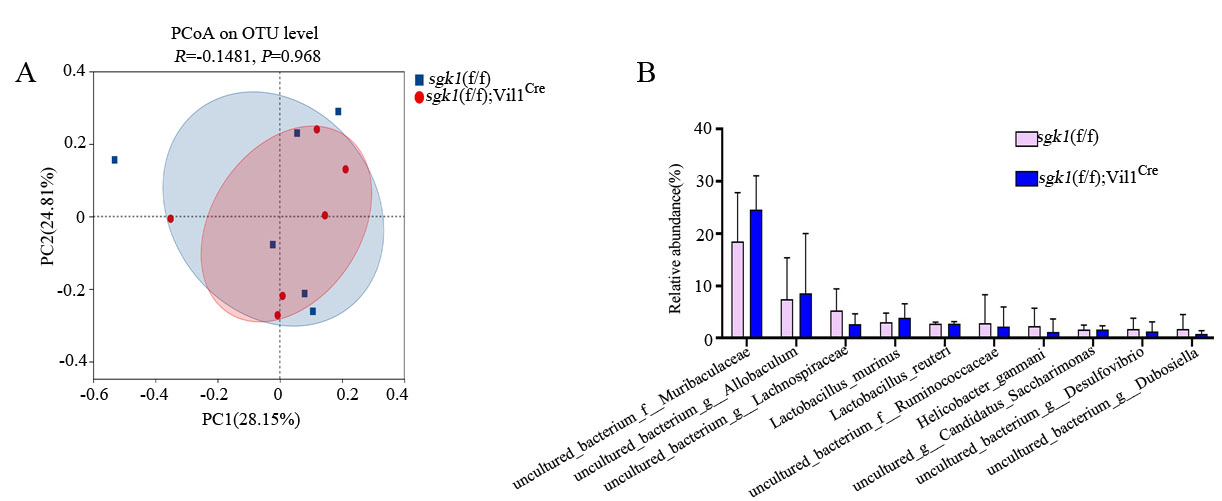


Supplementary Figure 4 Gut bacterial β-diversity based on principal coordinate analysis(A) and the relative abundance of main differential gut microbiota at species level (B) between mice with *sgk1*(f/f) and mice with *sgk1*(f/f);Vil1^Cre^ (8-10-week-old, 6 male mice per group) are shown.
